# Unveiling the Potential of Corn Cob Biochar: Analysis of Microstructure and Composition with Emphasis on Interaction with NO_2_

**DOI:** 10.3390/ma17010159

**Published:** 2023-12-28

**Authors:** Méghane Drané, Mohamed Zbair, Samar Hajjar-Garreau, Ludovic Josien, Laure Michelin, Simona Bennici, Lionel Limousy

**Affiliations:** 1 Université de Haute-Alsace, CNRS, IS2M UMR 7361, F-68100 Mulhouse, France; meghane.drane@uha.fr (M.D.); mohamed.zbair@uha.fr (M.Z.); samar.hajjar@uha.fr (S.H.-G.); ludovic.josien@uha.fr (L.J.); laure.michelin@uha.fr (L.M.); simona.bennici@uha.fr (S.B.); 2 Université de Strasbourg, F-67081 Strasbourg, France

**Keywords:** pyrolysis, biochar, material characterization, NO_2_ removal, adsorption

## Abstract

In the context of sustainable solutions, this study examines the pyrolysis process applied to corn cobs, with the aim of producing biochar and assessing its effectiveness in combating air pollution. In particular, it examines the influence of different pyrolysis temperatures on biochar properties. The results reveal a temperature-dependent trend in biochar yield, which peaks at 400 °C, accompanied by changes in elemental composition indicating increased stability and extended shelf life. In addition, high pyrolysis temperatures, above 400 °C, produce biochars with enlarged surfaces and improved pore structures. Notably, the highest pyrolysis temperature explored in this study is 600 °C, which significantly influences the observed properties of biochars. This study also explores the potential of biochar as an NO_2_ adsorbent, as identified by chemical interactions revealed by X-ray photoelectron spectroscopy (XPS) analysis. This research presents a promising and sustainable approach to tackling air pollution using corn cob biochar, providing insight into optimized production methods and its potential application as an effective NO_2_ adsorbent to improve air quality.

## 1. Introduction

Air quality involves monitoring and assessing the different types of pollutants in the atmosphere. In particular, nitrogen dioxide (NO_2_) is an important pollutant that is often subject to elimination and mitigation measures [1]. NO_2_ is a noxious gas emitted by a variety of sources, including automotive emissions, manufacturing processes, and the combustion of fossil fuels [2]. The presence of high NO_2_ concentrations in the air can cause serious health and environmental problems [3,4].

Improving air quality requires the implementation of actions to limit emissions of air quality contaminants such as NO_2_. This objective can be reached by employing cleaner technologies, tightening emissions regulations, developing renewable energy sources as well as fostering the use of sustainable transport technologies.

Among the technologies widely applied to remove NO_2_ gases are selective non-catalytic reduction (SNCR) and chemical scrubbing and selective catalytic reduction (SCR) [5]. Both SCR and SNCR function at gas temperatures in the 270–400 °C and 900–1000 °C range, respectively, suggesting that they would be most effectively operated in high-temperature flue gas settings [6,7,8,9,10]. However, the approach that is most appropriate for eliminating NO_2_ and mixed acids, e.g., nitric and phosphoric, involves wet washing with sodium sulfide and sodium hydroxide in accordance with the following equation: 8 NO_2_ + NaSH + 9 NaOH → 8 NaNO_2_ + Na_2_SO_4_ + 5 H_2_O [4]. Typically, sodium hydroxide is used in aluminum etching processes for the production of thin-film transistors and liquid crystal displays. However, the hydrogen sulfide odor is then emitted from the scrubber effluent and subsequently becomes a secondary pollution [10].

While selective catalytic reduction (SCR) has demonstrated high efficiency in removing or destroying nitrogen oxides (NOx), reaching around 90% [11], the process relies heavily on high reaction temperatures. In particular, typical catalysts, such as TiO_2_-supported V_2_O_5_-WO_3_ and V_2_O_5_-MoO_3_, as well as small-pore copper zeolites, are generally effective in the temperature range from 250 to 600 °C [11,12,13]. As a result, the abatement of NO_2_ emitted by low-temperature exhaust gases (<200 °C), as produced when vehicles are cold-started at the roadside [14], is a challenge. Indeed, such “over-emissions” of NO_2_ are ineligible with a total of 4.6 million tons, and in the European Union (EU-28) alone, they were associated with around 68,000 premature deaths due to NO_2_ pollution in 2016 [2]. Consequently, the development of effective new technology to mitigate ambient NO_2_ emissions becomes imperative.

Adsorption by means of solids-based adsorbents, a method that is based on interactions between the adsorbent and the adsorbate, is a promising solution for the elimination of NO_2_ [11]. The use of adsorbents offers advantages over conventional SCR catalysts, such as low-temperature versatility, opening significant prospects for adsorption technology [11]. The efficiency of NO_2_ removal by adsorption depends on the characteristics of the adsorbents. Various porous solid adsorbents, including zeolites [15,16], metal oxides [17], carbonaceous materials [18], silica [16], and metal-organic frameworks (MOFs) [19,20,21,22], have been investigated for capturing NO_2_ under ambient conditions. Nevertheless, just a few adsorbents have demonstrated a combination of high capacity, selectivity, and regenerability for NO_2_, mainly due to the high reactivity of NO_2_. Indeed, NO_2_ often reacts easily with MOF structures which are considered a promising adsorbent, leading to degradation of the structure by breaking coordination bonds. Furthermore, the NO_2_ adsorption process can produce nitric oxide (NO), a second toxic component of NO_x_, via a disproportionation reaction. The NO released in the process contributes to aggravating pollution issues worldwide. Therefore, it is essential to explore new adsorbents with both high NO_2_ removal efficiency and low NO release in order to achieve improved environmental performance.

In view of the acidic nature of NO_2_, adsorbent materials with a high density of base sites are expected to offer high NO_2_ capacity on the basis of acid-base interactions [2]. Biochar has been widely recognized for its inherent basic characteristics, and these properties have found a variety of applications. Among the most important is as a soil amendment, in which biochar’s ability to neutralize acidic soils is used to improve soil quality and promote optimum plant growth [23,24]. In addition, biochar has demonstrated its effectiveness as a buffering agent in the methanization process [25]. By making use of its inherent basicity, biochar helps to balance the process, thereby increasing biogas production. This dual role of biochar, both in soil improvement and in increasing biogas production thanks to its basic properties, underlines its versatile potential and impact in different fields.

Biochar materials can be regarded as promising materials for NO_2_ adsorption owing to a number of key considerations. Firstly, most biochar materials display basic properties owing to alkaline substances contained in the feedstock biomass [24]. Thus, alkaline sites present on the biochar surface, for example, alkali metal ions (potassium, sodium, calcium) or functional groups (hydroxyl groups) [26], are capable of interacting with acidic NO_2_ molecules via chemical reactions. These interactions imply acid-base reactions, whereby the biochar’s base sites neutralize the acidic nature of NO_2_, enabling it to be adsorbed and eliminated from the atmosphere [27]. In addition to increasing the adsorption capacity of NO_2_, the basicity of the biochar may also play a role in its selectivity towards NO_2_ compared with other gases [28]. Biochar’s base site affinity for NO_2_ enables preferential adsorption, effectively targeting and capturing this specific pollutant. Secondly, it has a highly porous structure offering a large surface area, thereby supplying numerous sites that can enhance its adsorption capacity for NO_2_ molecules [27]. Thirdly, it is comprised of carbonaceous materials from biomass, such as agricultural waste or forest residues [29]. Such biomass sources are plentiful and renewable, making biochar a sustainable option for NO_2_ adsorption when compared with conventional adsorbents. Finally, biochar is chemically reactive thanks to functional groups on its surface, including hydroxyl (-OH), carboxyl (-COOH), and phenolic groups [27]. Those functional groups can interact with NO_2_ molecules by adsorption mechanisms, thereby improving the efficiency of the overall adsorption [27].

The main objective of this innovative research is to conduct an in-depth investigation into the interaction between biochar and NO_2_ using advanced XPS analysis. This study represents a pioneering effort since it is the first exploration of the complex interaction between NO_2_ and biochar in the scientific literature. The main objective is to elucidate the mechanisms governing this interaction and, in turn, to design new biochars optimized for highly efficient NO_2_ sorption, thereby contributing significantly towards sustainable air pollution abatement policies. In order to realize this challenging goal, various biochars were prepared meticulously using corn cobs as feedstock. This selection is due not only to the abundance of corn cobs but also, and above all, to their intrinsic composition and properties [30,31], which make them ideal for the production of biochars suitable for NO_2_ sorption. Corn cobs, rich in cellulose, hemicellulose, and lignin, have a unique chemical composition which, during pyrolysis, favors the development of biochars characterized by complex porous structures and increased surface area. The aim of this approach is to evaluate the potential of corn cobs as a sustainable and environmentally friendly resource for biochar production. In addition, different pyrolysis temperatures were evaluated during biochar production, with the aim of comprehensively examining the influence of these temperatures on the physicochemical properties of the biochars obtained. The insights gained from this study will provide a fundamental basis for the design and development of tailored biochars designed specifically for superior NO_2_ sorption capabilities. Such innovative biochars can play a key role in mitigating air pollution, particularly the harmful effects of NO_2_ emissions. Using corn cob waste sustainably as feedstock for biochars also adds an ecological dimension to this research, contributing to the circular economy and waste recovery.

## 2. Materials and Methods

### 2.1. Preparation of Biochar

**Figure 1:**
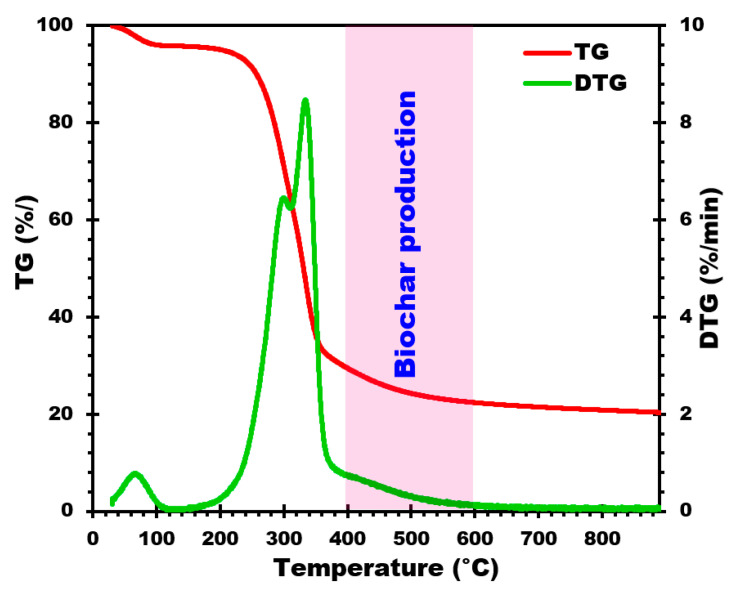
TG and DTG curves of corn cobs.

The raw material chosen for this study was corn cob (RM-CC) due to its abundance in the Alsace region of France. Prior to biochar production, RM-CC was ground using a BLIK knife mill to obtain a particle size fraction between 0.5 and 2 cm. The experiments to produce biochar from corn cobs were carried out in a pilot pyrolyser equipped with a screw conveyor to transport both the biomass (corn cobs) and the biochar through the pyrolysis reactor. In the course of the various pyrolysis, the gases produced (condensable and non-condensable fractions) are directed to a torchiere prior to complete combustion. At the furnace outlet, the biochars are recovered and transported in a double-walled tube equipped with a water-cooling system, enabling the temperature to be reduced to around 20 °C. Next, the biochars are stored in a hermetically sealed metal container, free of any trace of oxygen. Before introducing the raw material into the reactor, the entire pilot system is purged with a stream of nitrogen (20 NL/h) until the oxygen content in the reactor is below 1%vol. During the pyrolysis process, to ensure the absence of oxygen, the system is continuously flushed with nitrogen. The temperatures at which the biochars are produced, as determined by the ATG results presented in Figure 1 and discussed in Section 3.1, are 400, 450, 500, 550, and 600 °C, with a residence time of 30 min (equivalent to a feed rate of 2 kg/h) in the pyrolysis reactor. To facilitate reading the results associated with the different biochars, they will be designated as follows: B-CC-400, B-CC-450, B-CC-500, B-CC-550, and B-CC-600.

### 2.2. Techniques of Characterizations

A thermogravimetric analyzer (TGA 850 is a thermal gravimetric analyzer (TGA) manufactured by Mettler Toledo, Columbus, OH, USA) has been used to study the MR-CC thermal decomposition and determine its proximate analysis. In addition, the stability of the biochar produced at different temperatures and the determination of its proximate analysis were also studied using the same instrumentation. Prior to the experiments, the samples were dried overnight in a ventilated oven at 105 °C in the presence of air. In a typical run, around 40 mg of the RM-CC sample was accurately weighed and placed in an open-type alumina crucible (150 μL), which was subsequently placed in a TGA furnace. For this, the temperature setting had been increased from room temperature to 900 °C at a heating rate of 10 °C/min, with a continuous flow of nitrogen at 100 mL/min. This was kept at 900 °C for 10 min. After this, the atmosphere was changed to synthetic air (100 mL/min) for 60 min.

The parameters and conditions used to characterize the composites by X-ray Diffraction (XRD) analyses, X-ray Fluorescence, CO_2_ adsorption, and Scanning Electron Microscope (SEM) are detailed in our previously published papers [32,33,34].

The elemental analyses (CHONS, Cl) were conducted to determine the mass percentages of carbon [C], hydrogen [H], oxygen [O], nitrogen [N], sulfur [S], and chlorine [Cl] in both corn cobs and the produced biochars. These analyses were sub-contracted to the supplier Eurofins following the following standards: Sample preparation for analysis was carried out according to the NF EN ISO 14780 standard [35]. Elemental analyses for [C], [H], and [N] were performed according to the NF EN ISO 16948 standard [36], while the [Cl] and [S] contents were determined using the NF EN ISO 16994 (method A) standard [37]. The oxygen content was estimated through calculation (mass balance).

The porosity analysis of biochar using mercury was sub-contracted to the supplier FiLAB. They conducted the analysis using a Micrometrics Autopore IV instrument (Norcross, GA, USA). The sample was degassed for 3 h at room temperature and a pressure of 50 µmHg. Mercury intrusion was performed at 22 °C, with a contact angle of 130 °C, and a pressure range of 0.52 to 60,000 psia (0.036 to 4137 bar). The equilibration time was set to 10 s, covering a pore size range from 350 µm to 3 nm.

The pH and electrical conductivity (EC) of biochar were measured by mixing about 1 g of the biochar sample with 25 mL of deionized water. The pH measurement was conducted using an SI Analytics instrument Lab 845 (SI Analytics instrument, Mainz, Germany), while the EC measurement was performed with a pHenomenal instrument (PC 5000 L) (VWR, Radnor, PA, USA). After 24 h of stirring, the samples were centrifuged, and the pH and EC values were determined. To ensure accuracy, the pH meter was calibrated using buffers of pH 4, 7, and 10 prior to the measurements.

The characterization of produced biochar through Raman analysis was conducted using a Horiba Labram BX40 spectrometer (Horiba, Kyoto, Japan). The instrument featured a laser with a wavelength of 632 nm. To ensure biochar integrity, the laser power was reduced by a factor of 10 or 100 using a D1 filter placed along the optical path. This adjustment helped minimize fluorescence emission and potential phase transformations within the biochar. The spectrometer employed a holographic grating with 600 lines per millimeter, enabling precise dispersion and analysis of the Raman scattered light. The aperture size (Hola) was set at 1100 µm, while the slit size was adjusted to 500 µm. Raman spectra were collected within the range of 1600 cm^−1^, providing insights into molecular vibrations and structural characteristics of the biochar. A 50× magnification objective was utilized to focus the laser beam onto the biochar, allowing for detailed examination. Each Raman measurement lasted for a duration of 90 s, facilitating optimal signal accumulation and data acquisition for comprehensive analysis.

Biochar’s functional groups were analyzed using a technique called diffuse reflectance infrared Fourier transform spectroscopy (DRIFTS). For this analysis, we used a ThermoFisher Scientific Nicolet iS50 instrument (Waltham, MA, USA). DRIFTS measures the diffuse reflection of infrared radiation from a sample and provides information about the functional groups present on the biochar’s surface. One advantage of DRIFTS is its suitability for highly scattering and absorbing materials, making sample preparation less time-consuming. In the analysis, four radiation spectra were recorded for each biochar sample and for potassium bromide (2%) used as a reference. These spectra covered a range of frequencies from 4000 to 400 cm^−1^. From these radiation spectra, absorption spectra were calculated to determine the specific absorption patterns related to functional groups in the biochar.

X-ray photoelectron spectroscopy (XPS) measurements were conducted on a VG Scienta SES 2002 spectrometer located in Uppsala, Sweden. The spectrometer was equipped with a monochromatic Al Kα X-ray source, with Al Kα having an energy of 1486.6 eV. To compensate for the charging effect, an electron gun was employed. The XPS spectra were recorded using a pass energy of 100 eV for high-resolution scans and 500 eV for wide scans. The analysis chamber maintained a pressure of 10^−9^ mbar during the measurements. The analyzed zone had a surface area of 24 mm^2^ and an analysis depth of 9 nm, providing valuable information about the surface composition and properties. For calibration purposes, the binding energies (BEs) were calibrated using the C1s peak of carbon as the reference at 285 eV. To process the acquired spectra, the peaks were fitted using Gaussian-Lorentzian functions. This fitting process was carried out utilizing the XPS-CASA software (casaXPS software 2.3.18 Ltd., Teignmouth, UK). Prior to fitting, a Shirley-type background was subtracted from the spectra to enhance the accuracy of the analysis. The intensity area of each peak was determined by integrating the peak areas of individual components, taking into consideration various factors such as the cross-section, mean free path of an electron, and transmission function of the analyzer. These considerations play a crucial role in accurately quantifying the elemental composition and understanding the electronic structure of the analyzed samples.

### 2.3. NO_2_ Interaction with Biochar

To delve deeper into understanding the potential interactions between NO_2_ and the various biochars, a fixed-bed reactor setup was employed. This system involved depositing biochar onto a fused silica frit (40–100 µm) within a vertical quartz reactor, which had an internal diameter of 10 mm and extended 600 mm in length. Through this configuration, we facilitated continuous gas flow, enabling a more detailed examination of the interactions between NO_2_ and the biochar samples. This study focused on biochars produced under optimal conditions at temperatures of 450 °C, 500 °C, and 550 °C, representing the biochar samples tested. Each experiment involved 0.25 g of biochar samples. For the adsorption experiments, a precisely controlled NO_2_ concentration of 492 ppm was used to ensure test consistency. Experiments were conducted at a constant temperature of 20 °C, with each adsorption experiment taking one hour, allowing sufficient time for interactions between NO_2_ and biochars to occur. To maintain a constant gas flow in the reactor, the gas flow rate was set at 50 NL/h (Normal Liter per hour). In addition, the NO_2_ pressure was carefully adjusted to match atmospheric pressure during the experiments. After the adsorption tests, the biochar samples were recovered for further analysis using X-ray photoelectron spectroscopy (XPS). The XPS analysis aimed to explore the surface chemistry of the biochars and delve deeper into their specific interactions with NO_2_, highlighting potential mechanisms underlying the adsorption process.

## 3. Results and Discussion

### 3.1. Ultimate Analysis, Mineral Composition, and Proximate Analysis of Corn Cobs

Table 1 shows an analysis and comparison of the CHONS ultimate analysis and mineral composition of RM-CC, considered as the raw material intended for the preparation of biochar. The CHONS analysis gives the weight percentages of carbon (C), hydrogen (H), oxygen (O), nitrogen (N), and sulfur (S) on a dry basis. RM-CC used in this research had a composition of 48.00% C, 6.04% H, 43.00% O, 0.77% N, and 0.13% S. Upon comparing these values with references [30,38], we observe minor variations, which indicate a possible difference in RM-CC composition from different origins or processing routes. Moreover, RM-CC mineral composition has been examined. The analysis showed various elements, notably magnesium (Mg), aluminum (Al), silicon (Si), phosphorus (P), sodium (Na), chlorine (Cl), potassium (K), calcium (Ca), iron (Fe), zinc (Zn), and bromine (Br). Dry weight percentages of these elements were determined, with values of 0.051% for Mg and Al, 0.661% for Si, 0.108% for P, 0.015% for Na, 0.249% for Cl, 0.826% for K, 0.057% for Ca, 0.032% for Fe, 0.003% for Zn, and 0.002% for Br. When these values are compared with reference [39], there are slight variations in composition, which suggests potential differences regarding the mineral levels in RM-CC from various sources or treatment conditions.

Such findings can provide useful insights into RM-CC composition as a raw material for the production of biochar. The slight variations observed in both the final analysis and the mineral composition indicate that the properties of biochar obtained from RM-CC may vary according to the specific composition of the raw material.

To examine the thermal decomposition behavior of RM-CC, TGA-DTG (thermogravimetric analysis) was carried out under nitrogen flow. The TGA and DTG profiles are shown in Figure 1, respectively, illustrating the thermal decomposition curves and pyrolytic behavior of corn cobs during the pyrolysis process. In addition, this analysis also provides valuable insights into the thermal decomposition behavior of corn cobs during pyrolysis. The ranges of temperatures and peaks detected in the profiles are helpful in understanding the decomposition mechanisms of the different components of RM-CC, such as water, volatile matter, cellulose, and lignin [40]. These findings help in the optimization of pyrolysis conditions, thereby enabling biochar production with the targeted properties from RM-CC as a renewable biomass resource.

As shown in Figure 1, there is an initial loss of mass up to 150 °C, corresponding to water evaporation from the RM-CC sample [31]. The next step is a rapid decomposition stage between 200 °C and 360 °C, involving the release of volatile components [31]. This so-called active pyrolysis stage presents a significant peak at around 300 °C, pointing to cellulose decomposition [41]. Over 450 °C, a continuous slow decomposition stage can be observed, generally described as passive pyrolysis [24]. This corresponds to lignin decomposition leading to the formation of biochar [24]. Particularly, TGA-DTG profiles show only slight sample mass changes above 500 °C, which indicates that the decomposition process remains quite stable at higher temperatures.

On the basis of the DTG analysis (Figure 1) of the RM-CC, the optimum temperature range for biochar production is found to be between 400 and 600 °C. This is supported by the thermal decomposition behavior observed, and the pyrolytic characteristics exhibited by the RM-CC during the pyrolysis process. This conclusion is supported by the observed thermal degradation behavior and pyrolytic characteristics exhibited by the RM-CC during the pyrolysis process. By selecting this temperature window precisely (400, 450, 500, 550, and 600 °C), the pyrolysis of RM-CC can effectively lead to their decomposition, thereby producing biochar with suitable physico-chemical features. The temperature gradient chosen guarantees a controlled thermal decomposition process, allowing important physico-chemical characteristics of the biochar to be optimized. In this temperature window, it will be possible to promote the decomposition of organic components, which in turn will lead to the production of biochar with higher carbon content, greater stability, and a superior surface area. Such features are essential to enable biochar to be utilized in a broad field of applications.

The proximate analysis of RM-CC was determined based on TG on a wet basis (Table 2). The table compares the results of proximate analysis of RM-CC obtained in this study with the results provided in three published papers [40,42,43]. Among the studied components are moisture (Hm), volatile matter (VM), fixed carbon (FC), and ash.

In the present investigation, the average moisture content of RM-CC was found to be 4.33%. This is less than what has been documented in the literature (7.14%, 7.36%, and 11.7%, respectively) [40,42,43]. The lower moisture content in this study suggests that the RM-CC contained less water, possibly due to more efficient drying methods. The VM content was determined to be 75.75%. This is lower than what was stated by Shariff et al. [40] 87.76% and Demiral et al. [42] 79.58%, but higher than the value reported by Liu et al. [43] 69.5%. The variability in VM content may indicate differences in the organic compounds present in RM-CC, which may be due to differences in RM-CC types or treatment processes between studies. The FC content of RM-CC in this study was 18.64%. This is higher than the values reported by Shariff et al. [40] 11.19% and Demiral et al. [42] 11.57%, but lower than the value stated by Liu et al. [43] 15.9%. The FC represents the carbon-containing material remaining after the combustion of volatile materials. Differences in FC content from one study to another can be attributed to differences in RM-CC maturity, processing techniques, or analytical methods used. Ash content was established at 1.27% in this study, which is within the range of values described in the references (Ref [40]: 1.05%, Ref [42]: 1.49%, Ref [43]: 2.5%). The inorganic mineral content of RM-CC is represented by the ash content, and the discrepancies between the studies could be linked to differences in soil conditions, farming procedures, or analytical techniques. Compared with the references [40,42,43], the results of this study show considerably lower moisture and VM contents, higher FC content, and equivalent ash content. These discrepancies could be due to differences in RM-CC sources, harvesting methods, sample preparation, or analytical procedures used in the different research studies.

### 3.2. Biochar Characterization

#### 3.2.1. Biochar Yield

**Figure 2:**
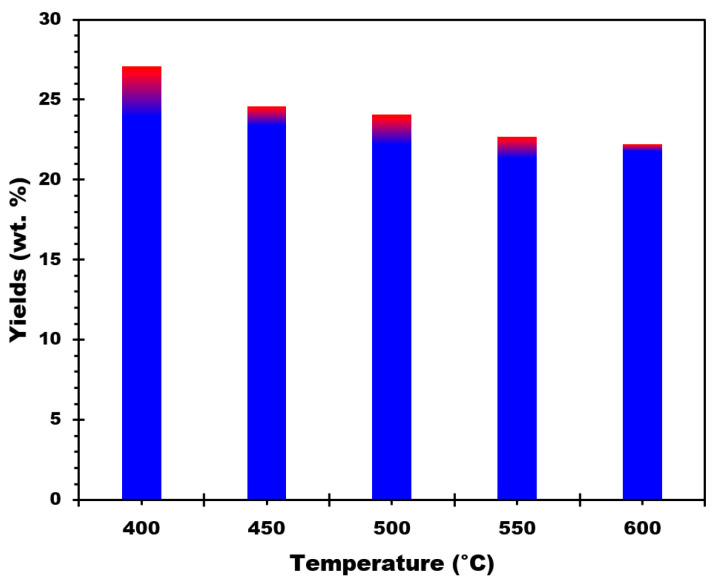
Biochar production yields for different pyrolysis temperatures.

Figure 2 shows the biochar yields obtained for RM-CC samples at different pyrolysis temperatures. The percentage of biochar produced relative to the biomass raw material is called biochar yield. The biochar yield was calculated using Equation (1):

(1) $$Biochar\;yield\;\left(\%\right)=\frac{m_{a}(g)}{m_{b}(g)}\times 100\%$$

where *m_a_* refers to the weight (g) of the sample after pyrolysis and *m_b_* is the weight (g) of the sample before pyrolysis.

The biochar yield was 27.08% at a pyrolysis temperature of 400 °C, according to the data. The biochar yield decreased slightly to 24.60% when the pyrolysis temperature increased to 450 °C. A temperature increase to 500 °C resulted in a slightly lower yield of 24.09%. The decline in biochar yields continues, with 22.26% at 550 °C and 22.22% at 600 °C. These results suggest that the pyrolysis temperature influences the biochar yield, with a gradual decrease as the temperature increases from 400 °C to 600 °C. Several conclusions can be derived from the biochar yield results at various pyrolysis temperatures:

- The maximum biochar yield is obtained at a pyrolysis temperature of 400 °C, with a yield of 27.08%.
- The biochar production is rather stable within a particular temperature range, implying that 400 °C is the temperature wherein the maximum transformation of RM-CC into biochar happens. The yield changes within a tight range of approximately 1.8% between 450 °C and 600 °C, ranging from 24.60% to 22.22%. This shows that at elevated temperatures, the biochar output reaches a rather constant state, demonstrating a constant conversion rate throughout this range of temperatures.
- The findings emphasize the significance of optimizing the pyrolysis temperature in order to achieve the target biochar production. Given the increased yield attained at that temperature, a temperature close to 400 °C may be preferred for maximizing biochar production efficiency.

#### 3.2.2. Ultimate Analysis, Mineral Composition, and Proximate Analysis of Biochars

Based on the results of the ultimate analysis performed on a dry basis, there were significant variations in the elemental composition of the biochars (Table 3). C content trended upwards with rising pyrolysis temperature, ranging from 48.00% in RM-CC to 87.30% in B-CC-600. Conversely, H content showed a decreasing trend, ranging from 6.04% in RM-CC to 1.82% in B-CC-600. Oxygen content also declined as pyrolysis temperature rose, going from 43.00% in RM-CC to 2.64% in B-CC-600. Both nitrogen and sulfur contents displayed comparatively small variations between the different biochars. These trends are attributed to dehydration and decarboxylation reactions, which are favored by the increase in pyrolysis temperature [44].

**Figure 3:**
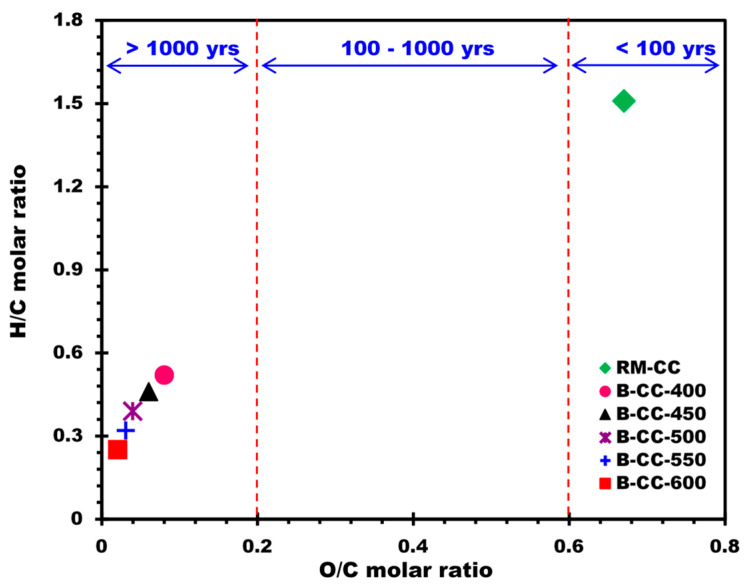
Van Krevelen graph of biochars prepared from corn cobs at different temperatures.

The Van Krevelen diagram (Figure 3) is frequently used in the analysis of the O/C and H/C molar ratios [45]. The H/C molar ratio, which is an indication of aromaticity and carbonization, decreased as pyrolysis temperature increased. For instance, the H/C molar ratio diminished from 1.51 in RM-CC to 0.25 in B-CC-600. This designates an increase in the aromaticity and graphitization of the biochars with higher temperature treatment. The diminishing H/C ratio suggests a higher carbon content comparative to hydrogen, inferring an increased carbonization process during pyrolysis. The O/C molar ratio, reflecting oxygenation degree, also showed a decreasing trend (O/C molar ratio decreased from 0.67 in RM-CC to 0.02 in B-CC-600), pointing to a decrease in oxygen functional groups in biochars as the temperature of pyrolysis increased.

These findings highlight the significant impact of pyrolysis temperature on the elemental composition and structural features of biochars. In particular, higher pyrolysis temperatures yielded biochars with higher carbon content, lower hydrogen and oxygen content, and reduced H/C and O/C molar ratios. Such variations in composition and elemental ratios may influence the physicochemical characteristics of biochars and their potential use in applications.

The O/C molar ratio results for the examined biochars revealed important information about their possible half-life. Previous research [46,47,48] has found a strong relationship between the O/C molar ratio and biochar half-life, demonstrating that different O/C ratio ranges correspond to varied half-life durations. According to the International Biochar Initiative (IBI), biochars with O/C ratios less than 0.2 are thought to be extremely stable and have a half-life of more than 1000 years [48,49,50]. With half-life ranging from 100 to 1000 years, biochars with O/C ratios between 0.2 and 0.6 are thought to be moderately stable. On the other side, biochars with O/C ratios larger than 0.6 have a half-life of fewer than 100 years, making them relatively unstable. The O/C ratios of the examined biochars ranged from 0.02 to 0.06, as evidenced by the findings in Figure 3. These O/C ratios fall within the range of highly stable biochars, indicating that the half-life of these biochars is probably greater than 1000 years.

Table 3 shows the mineral content of biochars made from corn cobs at various pyrolysis temperatures. The effect of pyrolysis temperature on the biochar formation process can be linked to the changes in element concentrations among the different biochars. The mineral composition of biochars and raw corn cobs (RM-CC) reveals the existence of alkali minerals and alkaline-earth minerals, which can alter the pH of biochars. As the pyrolysis temperature rises, several components’ concentrations change. For instance, concentrations of magnesium (Mg), aluminum (Al), and silicon (Si) often rise with rising pyrolysis temperatures. This is a result of the organic matter’s breakdown in RM-CC, which releases volatile substances while retaining relatively higher concentrations of minerals in the resulting biochars [51]. Alkali minerals such as K, Na, and alkaline-earth minerals such as Ca and Mg are commonly found in biochars. The alkalinity or acidity of these minerals can affect the pH of biochars [24,51]. As can be observed in Table 3, K and Na concentrations often increase as the pyrolysis temperature rises. K concentration, for instance, increases from 3.010 wt.% in B-CC-400 to 4.016 wt.% in B-CC-550. This is because, during pyrolysis, volatile substances are released and organic matter is thermally degraded, potentially leading to concentrations of alkali minerals in the biochars. Ca and Mg, both alkaline-earth minerals, show a rising tendency with increasing pyrolysis temperature. For example, Mg concentration rises from 0.315 wt.% in B-CC-400 to 0.475 wt.% in B-CC-550. These minerals are less volatile than alkali minerals and are more likely to stay in biochars during pyrolysis. The inclusion of alkaline-earth minerals may add to the biochars’ pH buffering capacity. The volatility and stability of different mineral compounds at different pyrolysis temperatures can also influence element concentration changes [24,51,52]. Some minerals may be more volatile and vulnerable to thermal breakdown, resulting in lower biochar concentrations, whilst others may be more stable and remain reasonably concentrated. Furthermore, the biochar synthesis formation involves physical and chemical transformations such as gas release, volatile compound condensation, and molecular structure rearrangement. These processes have the potential to alter the distribution and concentration of minerals within biochars, resulting in differences in element concentrations.

#### 3.2.3. pH and Electrical Conductivity Analysis

**Figure 4:**
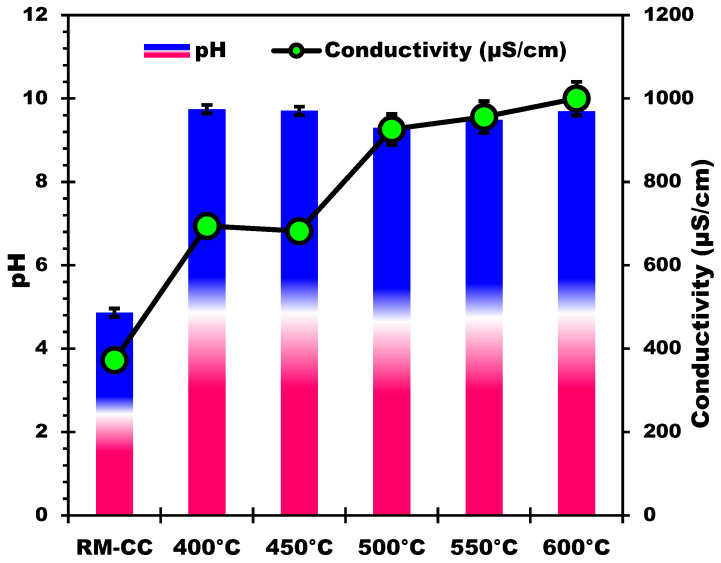
Temperature effect on pH and electrical conductivity of the produced biochars.

The measurement of pH and conductivity in relation to pyrolysis temperature offers useful information about the changes that occur during the thermal processing of biomass. As the pyrolysis temperature increases (Figure 4), there is a consistent rise in the pH values of the samples. This rise is attributed to a decrease in acidic functional groups like -COOH and -OH [53], alongside an increase in alkaline and alkaline earth minerals, released from the biomass and incorporated into the biochar during pyrolysis [47,54]. Higher temperatures boost the breakdown of organic components, resulting in the liberation of mineral elements, which contribute to the samples’ alkaline nature [55,56]. The observed pH increase suggests a move toward a more basic environment with greater pyrolysis temperatures. Similarly, as the pyrolysis temperature rises, the conductivity values rise as well (Figure 4). This tendency can be due to higher temperature samples having a higher concentration of dissolved ions. The release of mineral elements (such as potassium, calcium, magnesium, etc.), particularly alkaline and alkaline earth metals, increases the concentration of mobile ions in the solution, improving its electrical conductivity [56]. Higher conductivity values at higher temperatures support the presence and release of mineral elements during pyrolysis [47]. The mineral composition study of the samples adds to our understanding of the link between pH, conductivity, and mineral element concentrations. In accordance with the observed increase in pH and conductivity, the alkaline and alkaline-earth mineral composition rises as pyrolysis temperatures rise. The alkaline character of the samples and the resulting impact on pH values is caused by the release of components like magnesium, sodium, potassium, and calcium during pyrolysis.

#### 3.2.4. Thermogravimetric Analysis (TGA)

**Figure 5:**
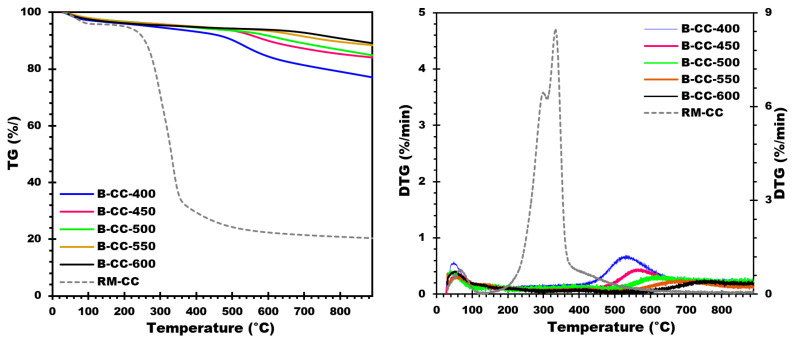
TG and DTG curves of biochar produced from corn cobs at different temperatures.

TGA analysis was performed under the same conditions as previously mentioned to validate the stability of the biochars generated and examine their effective pyrolysis temperatures. This TG-DTG analysis (Figure 5) gives useful information on the thermal behavior and decomposition of biochars. The TG examinations were carried out and the results are shown in Figure 5. The TG and DTG data (Figure 5) reveal that biochars generated from corn cobs are all stable at temperatures lower than their selected pyrolysis temperature. When the DTG thermogram was examined, it was discovered that each biochar had a single DTG peak. The peak occurred at temperatures of around 550, 570, 620, and 700 °C, with a little shoulder. This peak is caused by leftover lignin that was not entirely degraded throughout the pyrolysis process [24]. This peak was also influenced by the ash content of the biochars.

The proximate analysis in Table 4 illustrates changes in biochar derived from corn cobs across varying pyrolysis temperatures. As temperature increases, the percentage of VM decreases due to the heightened thermal breakdown of volatile organic molecules found in the raw biomass. This breakdown releases components like cellulose, hemicellulose, and lignin as gas or steam [55], resulting in reduced VM in the final biochar. Simultaneously, higher pyrolysis temperatures elevate the FC percentage by inducing carbonization and degrading complex organic compounds [47,55]. Consequently, this process generates more stable carbon structures within the biochar. Additionally, the ash content, representing inorganic mineral content from the biomass source, moderately increases with rising pyrolysis temperatures. The acceleration of breakdown and volatilization of organic components during higher temperatures leads to a biochar with a higher concentration of inorganic minerals (please refer to Table 3 of FX). Increasing pyrolysis temperature decreases VM while increasing FC in the biochar. This shift signifies the elimination of volatile organic components, resulting in a more carbon-rich and stable product [24]. The rise in ash content is attributed to the concentration of inorganic minerals present in the biomass feedstock during the pyrolysis process.

**Figure 6:**
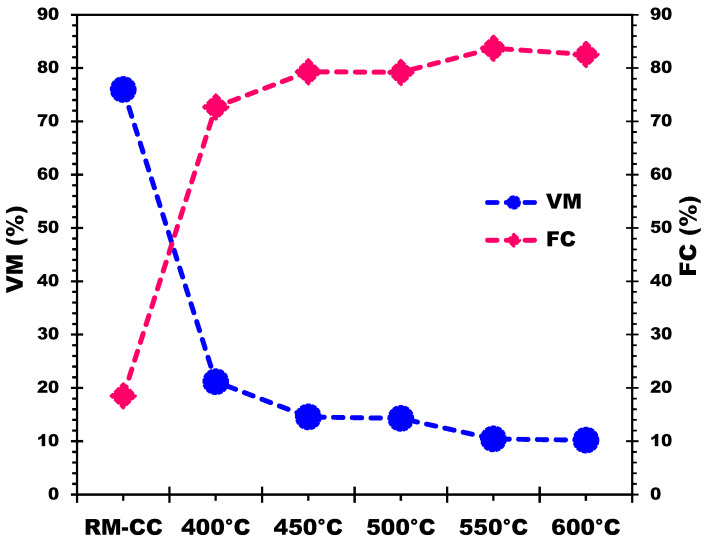
Effect of pyrolysis temperatures on VM and FC of the produced biochars.

Table 4 displays variations in VM and FC percentages with different pyrolysis temperatures, demonstrating an inverse relationship. The fluctuations are most significant at lower temperatures but diminish notably at 550 °C and 600 °C. Between 500 and 550 °C, VM decreases from 14.28% to 10.44%, while FC increases from 79.18% to 83.72%. Similarly, between 550 and 600 °C, VM reduces from 10.44% to 10.20%, while FC decreases from 83.72% to 82.55%. At temperatures of 550 °C and 600 °C, further temperature increases have a minor impact on VM reduction and FC increase, as depicted in Figure 6.

#### 3.2.5. Textural Properties of Biochars

**Figure 7:**
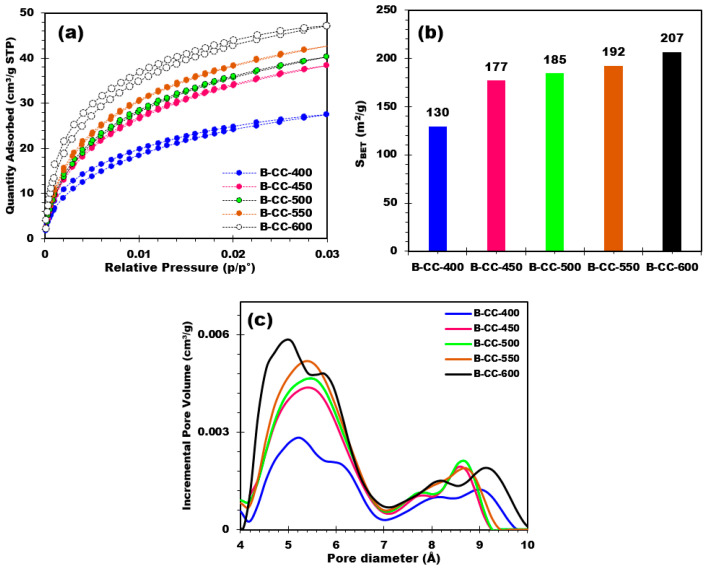
(**a**) Adsorption isotherms of CO_2_ at 0 °C, (**b**) S_BET_ of different produced biochars, and (**c**) pore diameter distribution for corn cobs-based biochars.

Analysis of the textural properties of biochars by CO_2_ adsorption makes it possible to evaluate the specific surface area (S_BET_), volume, and pore size distribution of biochars. The CO_2_ adsorption results show that increasing pyrolysis temperature leads to an increase in the amount of CO_2_ adsorbed by the biochars (Figure 7a).

The BET (Brunauer-Emmett-Teller) surface of biochar provides valuable insights into its porosity. An examination of the BET surface values of biochar produced at different temperatures reveals trends in surface development (Figure 7b). On the basis of Figure 7b, it is clear that there is an increase in biochar surface area as the production temperature rises. The BET surface area progressively rises from 130 m^2^/g for B-CC-400 to 207 m^2^/g for B-CC-600. This rise in surface area with temperature may be attributed to several factors. First, with higher temperatures, more thermal decomposition reactions take place, leading to the decomposition of organic constituents and the release of volatile materials. This process of breakdown creates extra pores and extends the total surface area of the biochar. As well as this, higher temperatures can promote pore formation, thus contributing to the increase in surface area.

Pore size distribution curves of the biochar samples showed different pore sizes (Figure 7c). Figure 7c demonstrates a progressive increase in pore volume with an increase in pore size, indicating a heterogeneous pore structure. Indeed, when comparing biochars prepared at different pyrolysis temperatures, there is a clear tendency towards increasing pore volume with increasing pyrolysis temperature. This trend is observed for all pore sizes. For lower temperatures, the incremental pore volumes are relatively smaller, pointing to a lower capacity for pore development. However, with increasing temperature, incremental pore volumes increase, which suggests more pore development and expansion. Indeed, the temperature effect on pore volume is especially pronounced in the small pore size range (4–7 Å). In this range, as the temperature increases, the incremental pore volume is greater, suggesting greater development of these small pores. Although in the higher pore size range (>7 Å), the influence of pyrolysis temperature on pore volume remains evident, while less noticeable in the lower pore size range. However, a progressive increase in pore volume is perceived with increasing pyrolysis temperature, signifying that higher temperatures as well contribute to the development and expansion of larger pore sizes. The pore volume evolution as a function of temperature reveals the influence of pyrolysis treatment on the development and expansion of biochar pores. The formation of both small and large pores is favored by higher temperatures, resulting in an increase in overall pore volume. This phenomenon can be attributed to thermal decomposition and volatilization of organic matter, leading to the creation of voids and pores in the biochar structure. As a conclusion, pore size distribution curves and pore volume evolution indicated that pyrolysis treatment temperature significantly affects pore development and expansion in biochar. These results underline the importance of temperature monitoring during biochar development to obtain the desirable pore characteristics and potentially improve biochar applications in fields such as adsorption, catalysis, energy, and soil improvement.

**Figure 8:**
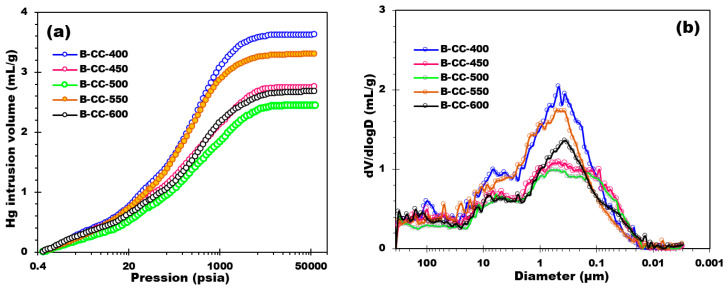
(**a**) Mercury intrusion volume curves of biochar samples and (**b**) Pore diameter distribution.

Figure 8 characterizes the results of mercury intrusion experiments carried out on prepared biochars. By analyzing Figure 8a, it is possible to perceive a distinct trend in the evolution of the biochars’ pore volume with increasing temperature. At lower pressures (e.g., below 10 psia), all the biochars display extremely low and analogous cumulative pore volumes, demonstrating minimal pore accessibility or intrusion of mercury. As the pressure increases, there is a gradual increase in the cumulative pore volumes for all the biochars. This put forward that higher pressures allow for more mercury intrusion into the pore structure of the biochars. Although as the pyrolysis temperature increases, a distinguished diminution in intrusion volume is perceived across the entire pressure range. This tendency shows that higher pyrolysis temperatures result in biochars with lower pore volumes and reduced porosity. The diminution in intrusion volume with increasing pyrolysis temperature can be attributed to the thermal degradation and alteration of the biochar’s organic components. Higher temperatures during pyrolysis cause stronger thermal decomposition reactions, leading to the formation of a denser carbonaceous matrix with reduced pore size and volume.

The pore diameter distribution (Figure 8b) of biochar synthesized at different temperatures discloses an extensive intrusion through a wide range of pore sizes, from 350 μm down to around 0.01 μm. The main mode of the pore size distribution is reliably determined to be approximately 0.46 μm, 0.46 μm, 0.55 μm, 0.44 μm, and 0.36 μm for biochars prepared at 400 °C, 450 °C, 500 °C, 550 °C, and 600 °C, respectively. It is noted that below 0.01 μm, compression phenomena of the material are possible. No extrusion is observed in any of the biochar samples. This suggests the presence of a network with a morphology of successive cavities that trap mercury. The average pore diameter distribution values for biochar samples produced at different temperatures are very similar indeed. In particular, the average pore diameters are around 32.18 μm, 32.18 μm, 32.17 μm, 32.17 μm, and 32.17 μm for biochars prepared at 400 °C, 450 °C, 500 °C, 550 °C, and 600 °C, respectively. From these values, it can be seen that the average pore size is relatively consistent across different temperature conditions, thereby indicating that the production temperature does not have a noticeable effect on the biochar’s average pore diameter. Overall, the pore diameter distributions of the biochar samples produced at diverse pyrolysis temperatures display similar profiles, with a wide distribution of pore sizes. Assuming completely cylindrical pores, the specific surface area of biochar generated at various temperatures was calculated by analyzing the incursion volume seen at each diameter. These results give an overview of the surface properties of the biochar samples. The changes in specific surface area between the temperatures examined, however, were not significant when analyzing macroporosity, with a percentage variance of less than 10%. At 400 °C, for instance, specific surface areas were 44.1 m^2^/g, 48.5 m^2^/g, 47.7 m^2^/g, 39.5 m^2^/g, and 48.1 m^2^/g. With only a minor percentage variation seen, these results indicate that temperature changes during biochar production had little impact on the macroporous surface of the material as determined by Hg prosimetry.

#### 3.2.6. Scanning Electron Microscopy Analysis

**Figure 9:**
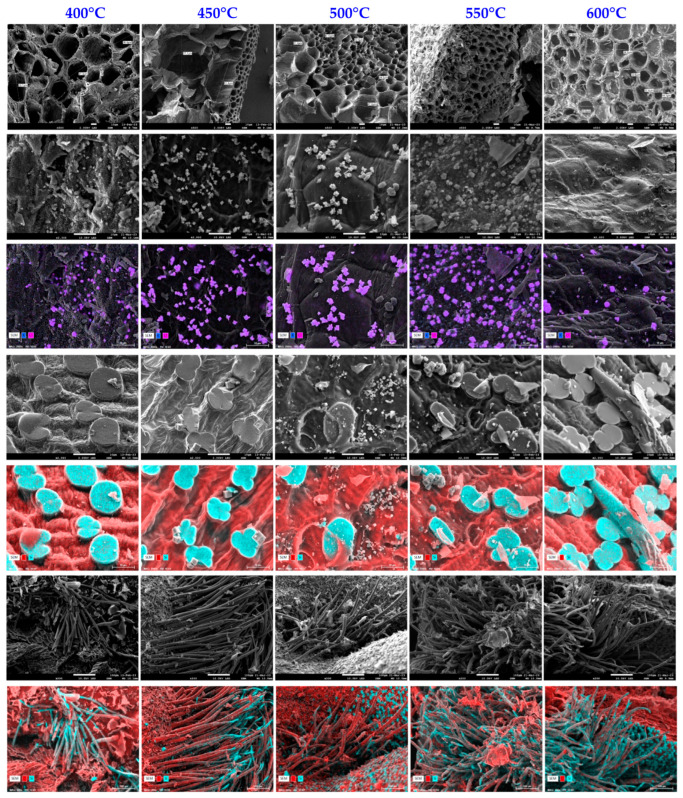
SEM and EDX images of biochar samples produced at different temperatures.

Surface analysis of biochars was conducted using SEM and EDX for high-resolution visualization of the surface of biochars as well as determination of the chemical composition and distribution of the elements present close to the surface (approximately 5 µm). To summarize, this technique enables microscopic inspection of the morphology and surface of biochars, as well as their chemical composition. SEM and EDX images (Figure 9) demonstrate that the biochars developed have a well-developed porous structure with open pores and porous walls.

These SEM and EDX photos were taken from several surfaces and show the existence of homogeneously scattered potassium chloride crystals in the shape of cubes, which are identifiable by the purple color. Furthermore, after pyrolysis, the corn cob structure retains its ultra-macroporosity, making it accessible to gaseous fluids without blockage during the pyrolysis or grinding stage. Images generated via EDX examination demonstrate the presence of silica phytoliths on the surface, which are identified by a blue color, while the material’s carbonaceous surface is depicted in red. Furthermore, SEM pictures reveal silica-coated carbon tubes, which are typical of corn cobs.

#### 3.2.7. Structural Analysis of Biochars

**Figure 10:**
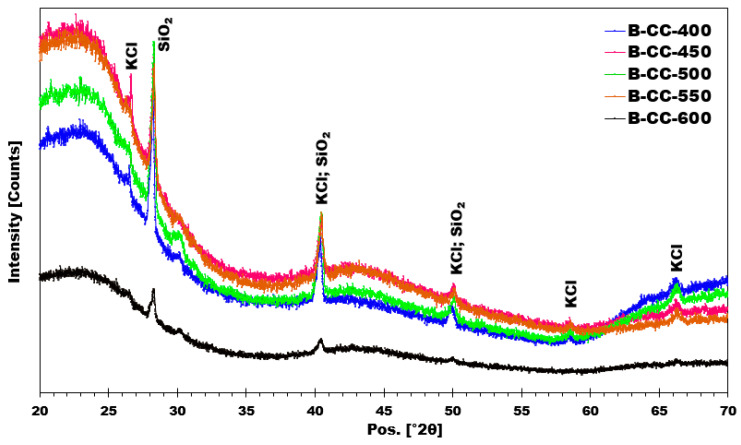
X-ray diffractograms obtained from the produced biochars.

X-ray diffraction (XRD) diffractograms for corn cob biochars (Figure 10) reveal a broad peak centered around 23°, corresponding to amorphous carbon. This observation is consistent with diffractograms observed for other biochars described in the literature [24]. It is also noted that all biochars produced at different temperatures exhibit similar crystalline phases, namely KCl and SiO_2_. These crystalline phases were present regardless of the pyrolysis temperature employed for biochar production. These findings suggest that the crystalline structure of biochars was not altered by increasing pyrolysis temperature.

**Figure 11:**
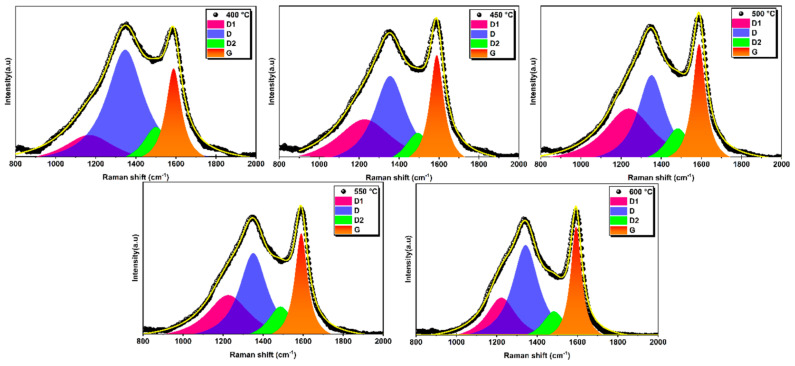
Raman spectra with deconvolution analysis of all prepared biochars.

The Raman spectra of the biochar samples are shown in Figure 11. In this figure, the D band around 1350 cm^−1^ and the G band around 1590 cm^−1^ can be observed for all biochars prepared at different temperatures. These bands correspond to amorphous and graphitic carbon, respectively [57,58].

Raman spectra of biochars can be decomposed into several individual peaks [59,60,61]. In this study, the original Raman spectra were fitted with four Lorentzian sub-peaks (bands D1, D, D2, and G) based on previous literature [59,60,62,63]. Figure 11 shows typical curve-fitting results for all biochars prepared at different temperatures. The D band, located near 1350 cm^−1^, is generally associated with various carbon structures. It is linked to disordered graphite structures, sp^2^ hybrid carbon atoms, aromatic rings, edge carbon atoms, and in-plane vibrations with structural defects [63,64]. This band indicates the presence of defects and disorder in the carbon structure. The D2 band, which originates from sp^2^-bonded amorphous carbon, fragments, or functional groups in the disordered structure, gives an indication of the specific functional groups present [65]. These functional groups can be carboxylic acids, phenols, ethers, and other oxygen-containing groups. The D1 band, associated with the sp^3^ structure and sp^2^-sp^3^ hybrid carbon near the microcrystal, gives indications of the types of carbon bonding present in the biochar [65,66]. This band may arise from the presence of sp^3^-hybridized carbon atoms and sp^2^-sp^3^ hybridization at the edges of carbon structures. It indicates the presence of graphite and non-graphite carbon domains. The G band, located near 1590 cm^−1^, represents the stretching vibration of aromatic rings and the crystal structure of sp^2^ carbon atoms [63,64,66]. It provides information on the graphitic carbon content and degree of graphitization of the biochar.

**Figure 12:**
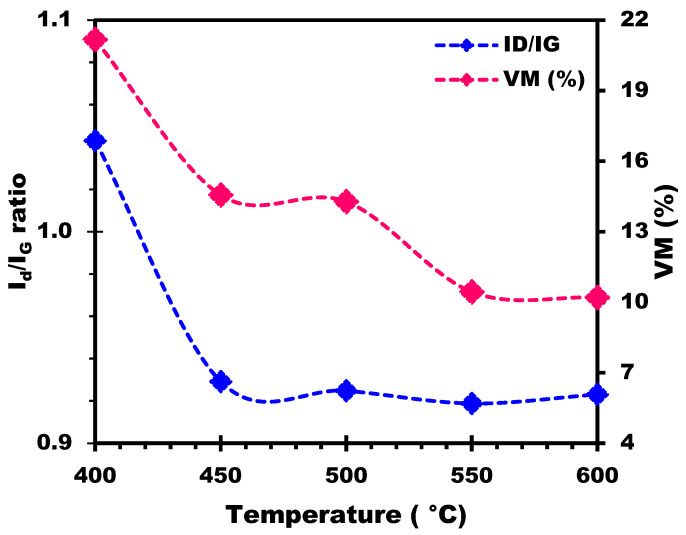
Influence of temperature on ID/IG ratio and its correlation with VM (%).

Investigating the evolution of the I_D_/I_G_ ratio in biochars produced at different temperatures can provide valuable information on their structural evolution and stability. From Figure 12, it can be seen that the I_D_/I_G_ ratio remains relatively constant within a narrow range when the temperature varies from 400 °C to 600 °C, with values of 1.043, 0.929, 0.925, 0.919, and 0.923 for temperatures of 400 °C, 450 °C, 500 °C, 550 °C, and 600 °C, respectively. Particularly at 400 °C, the biochar has a slightly higher I_D_/I_G_ ratio (1.043) than at the other temperatures, which indicates a relatively higher degree of disorder or the presence of defects in the carbon structure. This would suggest that the pyrolysis process at 400 °C produces a carbon structure that is slightly less graphitic and more disordered than at other temperatures. In addition, the decrease in the I_D_/I_G_ ratio indicates a decrease in the disordered or amorphous carbon content relative to the graphitic carbon content as temperature increases. This suggests that the pyrolysis process becomes more important at higher temperatures, causing an increase in carbon ordering and the formation of graphitic structures. The slight variations in the I_D_/I_G_ ratio between 450 °C and 600 °C indicate relatively stable structural changes in the biochar in this temperature range, with a percentage variation of just 0.65%. These minor variations could be attributed to slight variations in the carbon structure, the degree of carbonization, or the presence of defects in the biochar samples. Thus, biochars prepared at these temperatures exhibit similar levels of disorder or defects in their carbon structures, suggesting a relatively stable carbonization process and consistent biochar formation across the temperature range.

Moreover, as shown in Figure 12, a strong correlation was found between I_D_/I_G_ ratio trends and the percentage of volatile matter (VM). The higher I_D_/I_G_ ratio at 400 °C, indicating a higher degree of disorder or defects, can be associated with the higher VM content in the biochar prepared at this temperature. As the temperature rises, the pyrolysis process becomes more pronounced, resulting in the release and elimination of VM. This elimination of VM contributes to a decrease in the ID/IG ratio, indicating an increase in carbon ordering and the formation of graphitic structures. The relatively stable VM percentages between 450 °C and 600 °C, with no significant variations, suggest that the level of VM in biochar samples remains relatively constant over this temperature range. Consequently, the I_D_/I_G_ ratios also show similar stability, reflecting constant levels of disorder or defects in the carbon structures of the biochars.

### 3.3. Analysis of the Interaction between Biochar and NO_2_

The principal purpose of this sub-study is to investigate in detail the interaction between biochar and NO_2_ using XPS analysis. After thorough assessment, three biochars were selected for further study: biochar 450, biochar 500, and biochar 550. It is essential to emphasize that this study is the first of its kind in the scientific literature to focus on the interaction between biochar and NO_2_. It is a pioneering study aiming to elucidate the mechanisms and understand the complex interaction between biochar and NO_2_. The knowledge gained from this research will pave the way for the design of new biochars with suitable properties, particularly as adsorbents for NO_2_ sorption.

**Figure 13:**
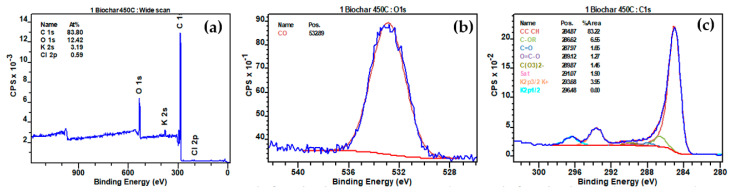
(**a**) Full range XPS spectra of biochar prepared at 450 °C before interaction with N_2_; (**b**) high-resolution fitted XPS O1s; (**c**) high-resolution fitted XPS C1s.

**Figure 14:**
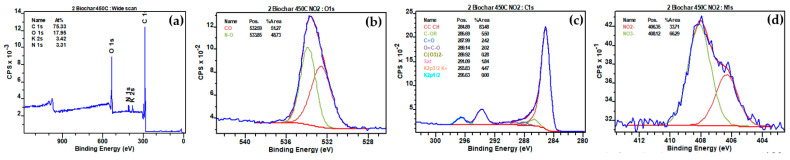
(**a**) Full range XPS spectra of biochar prepared at 450 °C after interaction with N_2_; (**b**) high-resolution fitted XPS O1s; (**c**) high-resolution fitted XPS C1s; (**d**) high-resolution fitted XPS N1s.

The XPS analyses of biochar produced at different temperatures (450 °C, 500 °C, and 550 °C) revealed significant peaks for O1s (C-O), C1s (CC CH, C-OR, C=O, O=C-O, C(O_3_)_2_^−^, Sat), K2p (K2p3/2 K^+^), and Cl2p (Cl2p3/2 Cl^−^) before interaction with NO_2_ (Figure 13, Figures S1 and S3, respectively). Following interaction with NO_2_, notable changes were observed in the XPS spectra (Figure 14, Figures S2 and S4). The reduction in the O1s (C-O) peak percentages were observed from 12.79% to 9.15%, 11.55% to 7.00%, and 11.56% to 6.09% for biochars produced at 450 °C, 500 °C, and 550 °C, respectively (Table 5), indicating a consistent decrease in carbon-oxygen bonds on the biochar surfaces after NO_2_ interaction. The interaction between NO_2_ and C-O bonds was depicted by the reaction equation: C-O + NO_2_ → CO + NO_3_^−^ [67], elucidating the cleavage of C-O bonds, the release of carbon monoxide (CO), and the incorporation of nitrogen-containing species in the form of nitrate (NO_3_^−^). Moreover, new N1s peaks at 406.35 eV and 408.11 eV, 406.26 eV and 408.11 eV, and 405.98 eV and 407.84 eV appeared for biochars produced at 450 °C, 500 °C, and 550 °C, respectively, indicative of nitrogen-oxygen bonds (NO_2_^−^) and nitrate (NO_3_^−^) presence post-interaction with NO_2_. The decline in K2p peaks, from 3.52% to 3.42%, 4.29% to 3.61%, and 4.35% to 3.94% for the respective biochars, suggested potential formations of K-NO_2_, signifying the chemical interaction of NO_2_ with potassium ions on the biochar surfaces (K^+^ + NO_2_ → K-NO_2_).

Based on the XPS results, we can propose a plausible mechanism for the interaction of NO_2_ with biochar. It is important to note that the following proposed mechanism is speculative and would require further experimental validation to confirm its accuracy.

Proposed Mechanism of NO_2_ Interaction with Biochar:

- Surface interaction: Initially, NO_2_ molecules adsorb onto the biochar surface by physical adsorption, weak van der Waals forces, or π-π interactions with aromatic carbon sites present in the biochar.
- Redox reactions: During interaction, some NO_2_ molecules may undergo redox reactions with specific functional groups on the biochar surface. This could lead to the formation of various surface-bound nitrogen species, such as nitro groups (-NO_2_) or nitrate groups (-NO_3_), as shown by the appearance of the N-O peak in the XPS spectra.
- Alteration of oxygen functionalities: The reduction of the CC CH peak in the XPS spectra suggests that interaction with NO_2_ may disrupt aromatic carbon-carbon bonds in the biochar structure. This may lead to the formation of oxygen-containing functional groups (e.g., carbonyl or carboxylic acid) on the biochar surface, as indicated by the appearance of the CO peak in the XPS spectra.
- K^+^ and NO_2_ interaction: The increased intensity of the K2p3/2 K^+^ peak after NO_2_ interaction may mean that NO_2_ is interacting with potassium-containing species present in the biochar. This interaction could be attributed to chemical reactions involving NO_2_ and potassium.

In general, the proposed mechanism suggests that NO_2_ adsorption on biochar implies a combination of physical adsorption, redox reactions, and alterations in surface chemistry. Both the formation of nitrogen-containing functional groups and changes in oxygen functionalities in the biochar structure reveal the existence of chemical interactions between NO_2_ and the biochar surface. More experimental studies, however, such as in situ studies and other surface analysis techniques, would be needed to validate and refine the suggested mechanism.

A future study, currently in progress, will go into more detail on the various experimental conditions, such as temperature, concentration, kinetics, etc. This detailed study will elucidate the adsorption capacities and behavior of biochar materials in different settings. An understanding of these interactions and mechanisms will be key to developing selective and efficient biochars as powerful adsorbents for NO_2_, thus making a significant contribution to the field of sustainable materials and the alleviation of air pollution.

## 4. Conclusions

In conclusion, this study highlights the significant influence of pyrolysis temperature on the properties of corn cob biochar. The research shows a decrease in biochar yield with increasing temperature. Elemental and mineral analysis, as well as O/C molar ratios, highlight the stability and high carbon content of biochars at higher temperatures. Textural and structural analyses indicate increased surface area and stable carbonization processes. Furthermore, the crucial finding of the chemical interaction between NO_2_ and biochar underscores the latter’s potential as a robust NO_2_ adsorbent. The biochar produced at 500 °C is particularly noteworthy, showing promising characteristics for interaction with NO_2_, as evidenced by substantial reduction of carbon-oxygen bonds, formation of nitrogen-oxygen bonds, and a consistent response to NO_2_ exposure. These results provide valuable insights for optimizing pyrolysis conditions to produce biochars suitable for various applications, such as environmental remediation, catalysis, and agricultural soil improvement. This study paves the way for further exploration of the potential of biochars to address environmental challenges and promote sustainable practices. Ongoing research will further investigate experimental conditions, including temperature, NO_2_ concentration, kinetics, and sorption capacities, to better understand biochar’s interactions with NO_2_ and its wider applications in sustainable development and environmental protection.

## Figures and Tables

**Table 1:** Analysis and comparison of ultimate analysis and mineral composition of corn cob raw materials.

CHONS Analysis (wt. %, Dry Basis)			
Element	Present Study	Ref. [30]	Ref. [38]
C	48	47	49
H	6.04	6.4	5.4
O	43	43.4	44.2
N	0.77	0.5	0.4
S	0.13	0.1	0
Mineral composition (wt.%, dry basis)			
Element	Present Study	Ref. [39]	
Mg	0.051	0.055	
Al	0.051	0.018	
Si	0.661	0.533	
P	0.108	0.111	
Na	0.015	0.01	
Cl	0.249	-	
K	0.826	1.038	
Ca	0.057	0.023	
Fe	0.032	0.008	
Zn	0.003	-	
Br	0.002	-	

**Table 2:** Proximate analysis of corn cob raw materials.

Proximate Analysis	This Study	Ref. [40]	Ref. [42]	Ref. [43]
(wt.%, Wet Basis)				
Humidity (Hm)	4.33	7.14	7.36	11.7
Volatile matter (VM)	75.75	87.76	79.58	69.5
Fixed carbon (FC)	18.64	11.19	11.57	15.9
Ash	1.27	1.05	1.49	2.5

**Table 3:** Analysis and comparison of ultimate analysis and mineral composition of biochars.

CHONS Analysis (wt.%, Dry Basis)						
Element	RM-CC	B-CC-400	B-CC-450	B-CC-500	B-CC-550	B-CC-600
C	48.00	80.50	82.20	84.60	87.00	87.30
H	6.04	3.53	3.17	2.80	2.33	1.82
O	43.00	9.03	6.81	4.98	3.02	2.64
N	0.77	0.87	0.75	0.88	0.79	0.86
S	0.13	0.12	0.17	0.11	0.11	0.13
H/C molar ratio	1.51	0.52	0.46	0.39	0.32	0.25
O/C molar ratio	0.67	0.08	0.06	0.04	0.03	0.02
Mineral Composition (wt.%, Dry Basis)						
Element	RM-CC	B-CC-400	B-CC-450	B-CC-500	B-CC-550	B-CC-600
Mg	0.051	0.315	0.404	0.326	0.475	0.381
Al	0.051	0.111	0.176	0.108	0.050	0.101
Si	0.661	1.102	1.411	1.170	0.725	1.149
P	0.108	0.313	0.382	0.350	0.331	0.367
Na	0.015	0.077	0.131	0.081	0.060	0.089
Cl	0.249	0.835	0.860	1.051	0.957	1.141
K	0.826	3.010	3.208	3.353	4.016	3.804
Ca	0.057	0.113	0.210	0.119	0.082	0.111
Fe	0.032	0.063	0.105	0.059	0.024	0.084
Zn	0.003	0.008	0.009	0.009	0.011	0.011
Br	0.002	0.003	0.004	0.006	0.0019	0.006

**Table 4:** Proximate analyses of prepared biochars from corn cobs.

	Proximate Analysis (wt.%, Wet Basis)			
	Humidity	VM	FC	Ash
RM-CC	4.17	75.96	18.51	1.36
B-CC-400	2.52	21.17	72.69	3.62
B-CC-450	1.92	14.56	79.31	4.22
B-CC-500	2.14	14.28	79.18	4.39
B-CC-550	1.96	10.44	83.72	4.64
B-CC-600	2.53	10.20	82.55	4.72

**Table 5:** XPS Results of Functional Groups in Biochar Before and After Interaction with NO_2_.

Biochar 450 °C				Biochar 450 °C after NO_2_ Adsorption			
Block Id	Name	Position	%At Conc	Block Id	Name	Position	%At Conc
O1s	C-O	532.8	12.79	O1s	C-O	532.5	9.15
C1s	CC CH	284.8	72.07	O1s	N-O	533.8	8.69
C1s	C-OR	286.6	5.68	C1s	CC CH	284.8	65.83
C1s	C=O	287.9	1.43	C1s	C-OR	286.6	4.34
C1s	O=C-O	289.1	1.10	C1s	C=O	287.9	1.91
C1s	C(O_3_)^2−^	289.8	1.26	C1s	O=C-O	289.1	1.59
C1s	Sat	291.0	1.64	C1s	C(O_3_)^2−^	289.9	0.22
K2p	K_2_p3/2 K^+^	293.6	3.52	C1s	Sat	291.0	1.45
Cl2p	Cl_2_p3/2 Cl^−^	199.1	0.61	K2p	K_2_p3/2 K^+^	293.8	3.42
				N1s	NO_2_^−^	406.3	1.11
				N1s	NO_3_^−^	408.1	2.19
Biochar 500 °C				Biochar 500 °C after NO_2_ Adsorption			
Block Id	Name	Position	%At Conc	Block Id	Name	Position	%At Conc
O1s	C-O	532.7	11.55	O1s	C-O	532.5	7.00
C1s	CC CH	284.8	73.67	O1s	N-O	533.7	8.46
C1s	C-OR	286.6	3.90	C1s	CC CH	284.8	68.73
C1s	C=O	287.9	1.61	C1s	C-OR	286.6	3.99
C1s	O=C-O	289.1	0.94	C1s	C=O	287.9	1.63
C1s	C(O_3_)^2−^	289.9	1.70	C1s	O=C-O	289.1	1.53
C1s	Sat	291.1	1.68	C1s	C(O_3_)^2−^	289.9	0.33
K2p	K_2_p3/2 K^+^	293.7	4.29	C1s	Sat	291.0	1.51
Cl2p	Cl_2_p3/2 Cl^−^	199.2	0.66	K2p	K_2_p3/2 K^+^	293.8	3.61
				N1s	NO_2_^−^	406.2	1.05
				N1s	NO_3_^−^	408.1	2.15
Biochar 550 °C				Biochar 550 °C after NO_2_ Adsorption			
Block Id	Name	Position	%At Conc	Block Id	Name	Position	%At Conc
O1s	C-O	532.7	11.56	O1s	C-O	532.14	6.09
C1s	CC CH	284.8	73.90	O1s	N-O	533.45	9.27
C1s	C-OR	286.6	3.61	C1s	CC CH	284.44	70.64
C1s	C=O	287.9	1.49	C1s	C-OR	286.22	2.35
C1s	O=C-O	289.1	1.04	C1s	C=O	287.54	1.22
C1s	C(O_3_)^2−^	289.9	1.68	C1s	O=C-O	288.69	1.34
C1s	Sat	291.1	1.63	C1s	C(O_3_)^2−^	289.54	0.23
K2p	K_2_p3/2 K^+^	293.7	4.35	C1s	Sat	290.64	1.56
Cl2p	Cl_2_p3/2 Cl^−^	199.2	0.74	C1s	K2p3/2 K^+^	293.57	3.94
				N1s	NO_2_^−^	405.98	0.86
				N1s	NO_3_^−^	407.84	2.51

## Data Availability

Data are contained within the article and Supplementary Materials.

## Supplementary Materials

The following supporting information can be downloaded at https://www.mdpi.com/article/10.3390/ma17010159/s1. Figure S1: XPS spectra of biochar prepared at 500 °C before interaction with N_2_. Figure S2: XPS spectra of biochar prepared at 500 °C after interaction with N_2_. Figure S3: XPS spectra of biochar prepared at 550 °C before interaction with N_2_. Figure S4: XPS spectra of biochar prepared at 550 °C after interaction with N_2_.
