# Supplementary material for: Unveiling the Potential of Corn Cob Biochar: Analysis of Microstructure and Composition with Emphasis on Interaction with NO2
Source: Materials (Basel). 2023 Dec 28;17(1):159. doi: 10.3390/ma17010159 (PMC10780219; doi:10.3390/ma17010159)
Supplement: Supplementary file 1 [file materials-17-00159-s001.zip › materials-2772177-supplementary.pdf]

<u>Supplementary Materials</u>

Unveiling the potential of corncob biochar: Analysis of microstructure and composition with emphasis on interaction with NO_2

Méghane Drané[1,2], Mohamed Zbair[1,2], Samar Hajjar-Garreau[1,2], Ludovic Josien[1,2], Laure Michelin[1,2], Simona Bennici[1,2], Lionel Limousy [1,2,*]

[1] Université de Haute-alsace, CNRS, IS2M UMR 7361, F-68100 Mulhouse, France ;
[2] Université de Strasbourg, France
* Correspondence: lionel.limousy@uha.fr ; Tel.: +33 (0)3 89 60 87 05

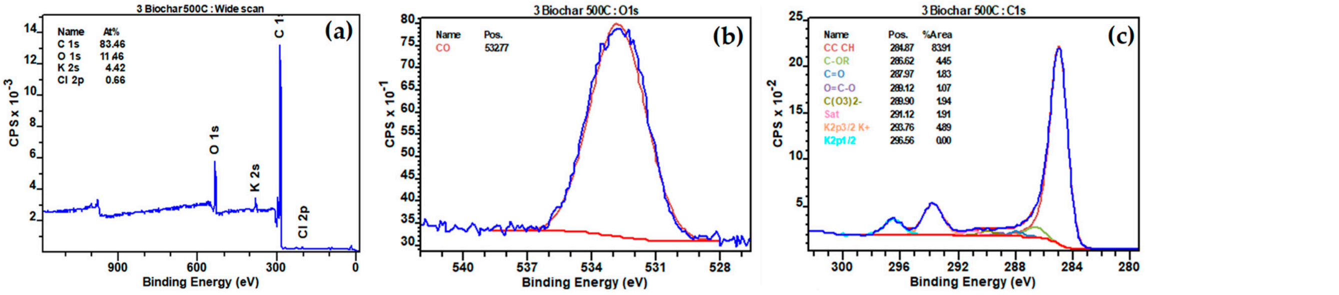

Figure S1. (a) Full range XPS spectra of biochar prepared at 500°C before interaction with N_2; **(b)** high-resolution fitted XPS O1s; **(c)** high-resolution fitted XPS C1s.

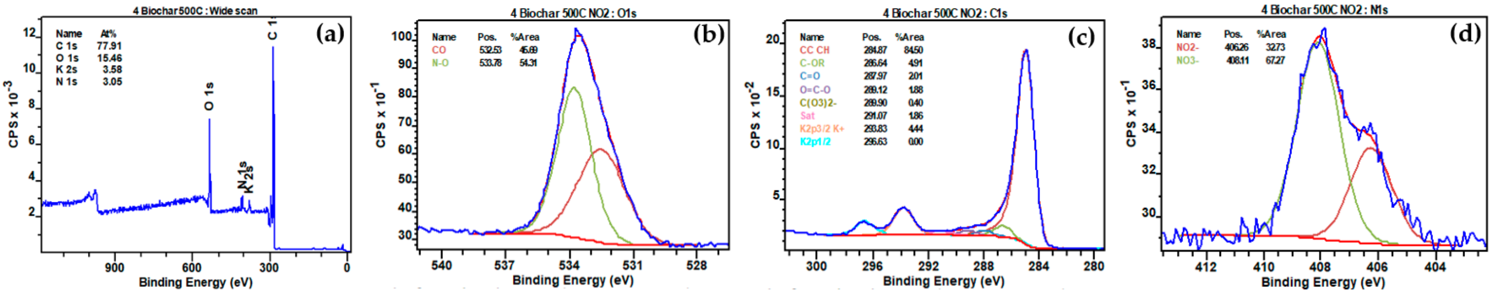

Figure S2. (a) Full range XPS spectra of biochar prepared at 500°C after interaction with N_2; **(b)** high-resolution fitted XPS O1s; **(c)** high-resolution fitted XPS C1s; **(d)** high-resolution fitted XPS N1s.

Supplementary Materials

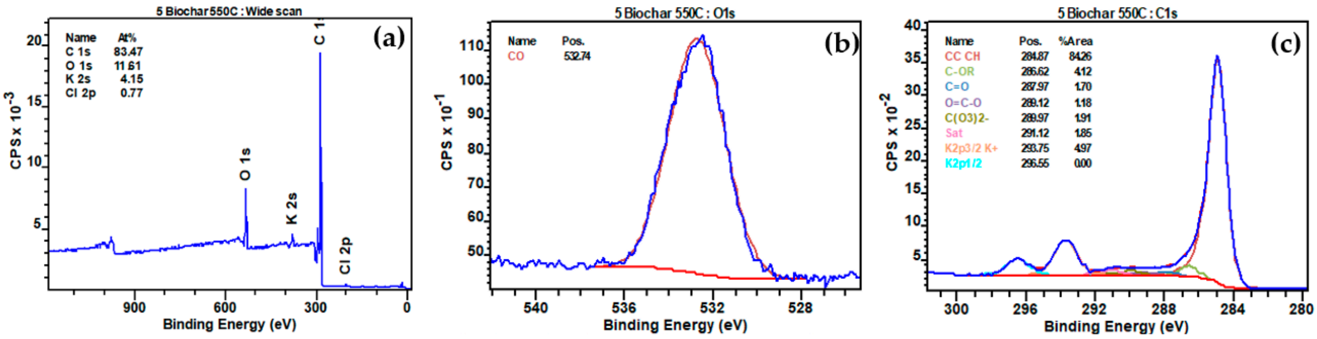

Figure S3. **(a)** Full range XPS spectra of biochar prepared at 550°C before interaction with N_2; **(b)** high-resolution fitted XPS O1s; **(c)** high-resolution fitted XPS C1s.

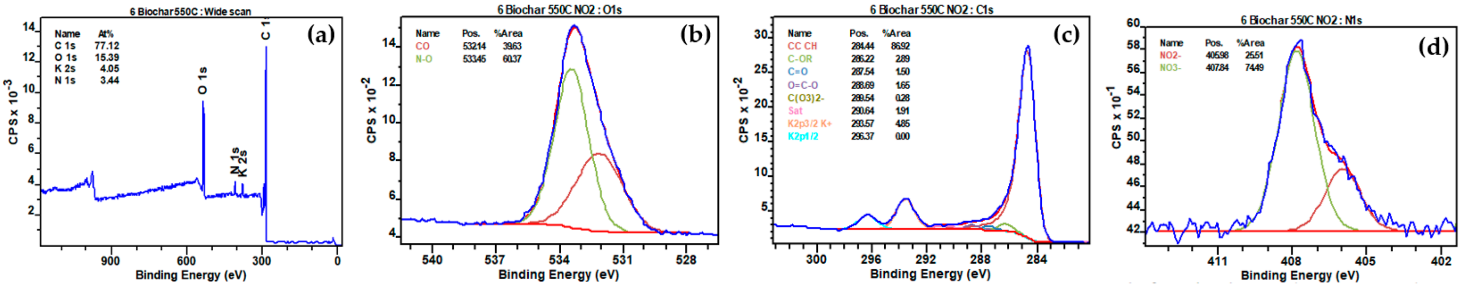

Figure S4. **(a)** Full range XPS spectra of biochar prepared at 550°C after interaction with N_2; **(b)** high-resolution fitted XPS O1s; **(c)** high-resolution fitted XPS C1s; **(d)** high-resolution fitted XPS N1s.
